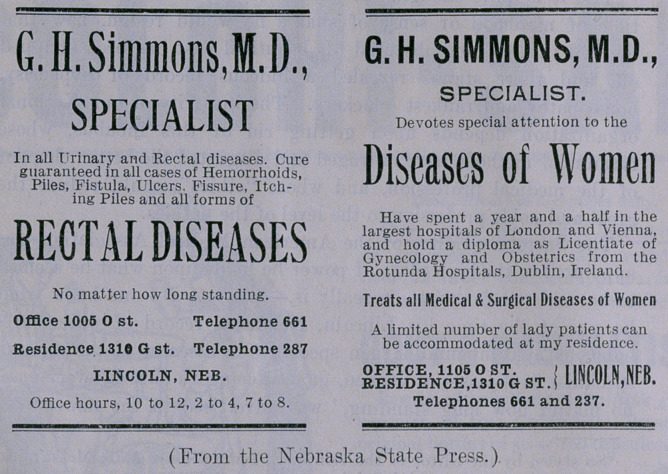# The Lifting of the Veil

**Published:** 1909-05

**Authors:** 


### THE LIFTING OF THE VEIL.

"Ye would be dupes and victims and ye are.
Is it enough? Or must I, while a thrill
Lives in your sapient bosoms, cheat you still?"
—*Lalla Rookh.*

**Our Peerless Leader.** **A Modern Mokanna**

Yes, it is quite enough. If "Our Peerless Leader"* had a particle of manhood or sense of shame he would resign, now that the veil that so long obscured his beautiful past has been stripped off, and there stands revealed a hideous record of hypocrisy, homeopathy and rankest quackery. The integrity of our National organization depends upon getting rid of this incubus, whose tyrannical methods have outraged and disgusted the better element of the medical profession, and whose "ethics" would reduce the great and noble profession to the level of the pathies.

The Secretary-Editor of the American Medical Association was appointed to the high place of power he holds upon what he seemed to be, and not for what he really is,—or was. No one knew what he was at the time his Lincoln, Nebraska, record of water-cure-homeopathy-compound-oxygen specialist in diseases of the rectum, specialist in diseases of women, guaranteeing "a cure in every case, no matter how long standing," was advertised in the newspapers

*So styled by the editor of the Kentucky Tentacle, one A. T. McCormick, who jointly with the Chief Spieler was pardoned when indicted for violating the sanitary laws of Kentucky. I have a *fac simile* of the pardon. —D.

afte rthe manner of the charletan and quack. Had this been known, does any one suppose for a moment that he would have been appointed to succeed the great Hamilton and the greater Davis? The veil of twenty years—ten or more of pretended "regularity"—which obscured the hideous past has been ruthlessly stripped off, as was that of pretended holiness of the False Prophet of Khorassan, Mokanna, who had so long deluded his fanatical followers, and he stands revealed in a record of all that legitimate practitioners of rational medicine hold in abhorrence,—as hideous as was the face of Mokanna,—so shocking that Zelica, his "Bride of Heaven," swooned on beholding it.

In addition to the Lincoln Medical Institute and water-cure-compound-oxygen advertisement in the Nebraska papers, which I reproduced in the January "Red Back," the "Peerless Leader" was running the two ads herewith reproduced. These are the latest findings from the graveyard of dead quackery, unearthed at Lincoln, Nebraska. Read them: "A cure guaranteed in every case, no matter how long standing," and "Have spent a year and a half in the largest hospitals of London and Vienna," and "Hold a diploma as Licentiate of Gynecology and Obstetrics from the Rotunda Hospitals of Dublin, Ireland." "A limited number of lady patients can be accommodated at my residence." Shades of Briggs, go blush!

Professor G. Frank Lydston, of the Illinois Medical College, has issued several pamphlets in which he asserts and presents documentary evidence to prove that Dr. Simmons "matriculated by proxy at Rush Medical College, Chicago," in the fall of 1891, and received a diploma from that school in March, 1892, a large part of which time he was practicing homeopathy in Lincoln, Nebraska, more than three hundred miles away. This Dr. Lydston substantiates by photographic reproductions of death certificates, with dates, and prescriptions with dates ("diphtheria" spelled "diptheria," and one prescription for "Firwein," whatever that is), and all the record Dr. Lydston could find of "Our Peerless Leader's" stay in Chicago (there was none at Rush) covered a period of twenty-two days—ten one time and twelve at another. Well may Dr. L. ask, "Was Rush a diploma mill in 1891-92?" Charges based upon this evidence have been brought against Dr. Simmons in the Chicago Medical Society and in the Illinois State Medical Association.

(A copy of Lydston's pamphlets can be had for the asking.)

"And this is the man who is the head and front of American medical journalism; who dictates our policies, controls medico-political appointments, supervises our organization, censors our articles, handles the business of our great journal, tells us what there is of value in our armamentarium therapeuticum, tells us what shall be advertised and what shall not, supervises the ethics and morals of our drug manufacturers, tells our independent medical journals what they shall advertise and what they shall not; receives invitations to lecture before our medical societies on the proper methods of teaching therapeutics—in short, who is the arbiter of all things literary, ethical, political, therapeutic and moral in American medicine."—*Lydston.*

And this is the man and this is the conduct that is defended by the controlled press,—the collar editors like Chase, and the Kentucky "Mac" who edits the Kentucky Tentacle—a brother of "Pardoned" Mac-the-Mick, chief spieler and walking delegate; and the Indiana personage, one Bullar, I think his name is, who runs the Hoosier State Tentacle. Will the reputable element of American medicine stand for it?

---

JUSTICE.—W. B. Atkinson. This venerable man was for many years the honored, respected and loved Secretary of the A. M. A. Many a doctor who reads these words will remember his kindly ways, his unfailing courtesy—a man who never could find it in his heart to refuse a favor to anybody. Dr. Atkinson is now living in comparative poverty, in advanced age. It would be a kindly act to pension the deserving old Secretary for his few remaining years. The treasury of the A. M. A. is full to overflowing with the voluntary contributions of thousands of physicians. We could well afford to grant him one hundred dollars a month, in recognition of the services he rendered us during the many lean years, when his personal influence meant much to the struggling association. Were such a proposition to be made in the general meeting of the Association, it would show how its justice was appreciated. Shall it not be made?—*Medical Standard.*—[And,—he was turned down for—Simmons.]

### A Critic Criticised—A Literary Bull in a China Chop.

Dr. Ralcy Husted Bell has just published a somewhat remarkable book entitled *The Changing Values of English Speech,** a copy of which is before us. There are many defects in it, but nevertheless, it is well written and is of absorbing interest.

Dr. Bell is the author of *The Worth of Words,* and of a volume of poems. He has the reputation of being a scholar, and, it seems, has made a special study of philology. He might even be called a genius, in a sense, and hence it is a matter of great surprise that in this book he shows a lack of knowledge of one, at least, of the most famous and familiar classic myths, that of the *Danaides.* In attempting to account for the name "Albion," the early name of Britain, he says:

"There is an English romance which tells us how badly the fifty sons of Danus, King of Greece, were treated by their wives, the fifty daughters of Aegistus, King of Greece. These faithless spouses thirsted for power. The question as to who should be 'boss' was uppermost in their minds. They hatched a murderous conspiracy by which they hoped to slay their husbands and to rule in their stead. One of their own sisters, as usual, betrayed them. Thereupon they were seized and set adrift in ships upon the sea. After many days of stormy weather, they landed in safety upon the shores of a large island, which was uninhabited. Upon this land they made their home and called it 'Albion' in honor of Albina, their eldest sister."

Now, this is rank nonsense. I scarcely feel like seriously criticising such stuff, and should not do so, perhaps, but for the fact

*Hinds, Noble & Eldridge, New York.

that he criticises Professor Lounsbury for saying: "There are matters in regard to which no height of genius can supply the place of a little accurate knowledge." Dr. Bell takes issue with him and implies, at least, that lack of knowledge doesn't count, and that form and style and rules of grammar are everything, and that syntax is of paramount importance. He illustrates and makes good his contention, in the foregoing, in which he shows a woeful "lack of accurate knowledge," for his syntax is all right, and his "form" and "style" are good.

The King was not Danus, but *Danaüs,* and he was not King of Greece, but of Lybia, first, and later of Argos. The story he attempts to tell is not "romance" but classic. Danaüs *had no sons,* but had fifty *daughters,* the famous Danaides; and his brother, Aegyptus (not "Aegistus") had no *daughters,* but had fifty *sons.* These fifty sons, with their father, Aegyptus, so persecuted Danaüs that, with his daughters, he fled to Argos. The king, one Gelener, abdicated in his favor because he brought the arts of civilization with him, and he became king of that country. His brother, Aegyptus, later king of Egypt, followed him with peace offerings, and they patched up their quarrel, and to cement the friendship the fifty sons of Aegyptus married the fifty daughters of Danaüs. But Danaüs being suspicious of treachery, followed what, in these days, we call David Harum's version of the Golden Rule: "Do unto the other fellow what he would do to you, but do it first." He induced the brides to assassinate their husbands on the wedding night, giving each one of them a dagger for that purpose. All the daughters obeyed him and slew their spouses, except Hypermnestra, who spared Lynceus, her husband, "for the delicate regard he had shown her modesty." For this disobedience her father imprisoned, but later forgave her. But the others were, nevertheless, punished by the gods in the underworld, where they were condemned to draw water, forever, in perforated vessels. I thought every schoolboy knew this fable, and its significance,—drought, thirsty soil and springs in the desert.

From the union of Hypermnestra and Lynceus descended in successive generations Abas, Acrisius, Danae, the mother of Perseus by Jupiter in the guise of a "shower of gold"; and Perseus, by Andromeda, begat Perses, Electryon and Alcaeus, and Alcmene was the daughter of Electryon, and by her Jupiter begat Hercules. (This is very like teaching a kindergarten.) If all those bad girls had been shipped off to Britain as Bell says they were, we should never have had the delightful fables of Perseus and

Andromeda, nor of Hercules and Alcmene, his mother, who, not being able to suckle him, prevailed on Juno to be his wet nurse. The young scamp bit her, when she jerked the breast away, spilling the milk across the sky, giving us the Milky Way (Galaxia). Bell is thus, unconsciously, the greatest iconoclast of any time. You see, we would have had no Milky Way! Think of that!

Dr. Bell's attempt to trace the name "Britain" to one Brutus, or "Bru," son of Silvius, son of Ascanius, son of Aeneas," is still more absurd, and displays ignorance of the subject even more than in the foregoing bosh. In the first place, Sylvius (S. Posthumus, not "Silvius" as Dr. Bell calls him) was not the *son* of Ascanius, but the half-brother, being the son of Aeneas by Lavinia, the Latin princess, daughter of Latinus, from whom and Aeneas sprang the Latin race; while Ascanius was the son of Aeneas by Creüsa, daughter of Priam, King of Troy, who, with the little boy Ascanius, fled from burning Troy, when, as Virgil says, "Aeneas from the flames of Troy the tired Anchises bore." His name was changed to Iulus, when at the end of his wanderings Aeneas, with the aid of old Evander, the exiled Arcadian king, had conquered the Latins, and cutting Turnus out, married the daughter, Lavinia. Hence the story of "Brutus, a so not Silvius," having discovered Britain is a pure fabrication. Aeneas founded Lavinium. Ascanius founded Alba Longa, "the birthplace of Romulus and Remus and the cradle of Rome" (Gayley). Aeneas had been told by Helenus and the river-god to build the city where he would find a *white* sow with thirty pigs lying down. This was done (B. C. 1152). I don't know what the sow and pigs had to do with it, other than to suggest "Alba." It was a *white* sow, mind you. But it seems that in those days it was the practice to regard the prophecies of alleged oracles as omens or portents, and to select the sites for cities in accordance with them. For instance, Ilium (Troy) and Thebes in Boetia, I believe, were built where a cow or a heifer laid down. Moreover, Alba was built on the exact spot where Aeneas first landed and found the old Arcadian, Evander, in his little scattering village, when he ascended the river in the first boats that had ever disturbed old Father Tiber, the deity of that famous stream and "Presidin' Elder of the dee-strict."

The first Brutus of whom we have any record is Brutus I., grandson of old Tarquin the Proud, last King of Rome. Old T. was so cruel that Brutus, having seen his father and brothers slain by him, was afraid of him, and he affected imbecility, and pretended that he could not speak. For this reason he was called

"Brute." An oracle when asked when the Tarquin dynasty would end, replied, "When the dog shall speak with human tongue." This prophecy was literally fulfilled. Tarquin Sextus, son of the old king, raped Lucretia, the wife of Colatinus, a relative of B., and Brutus raised a revolution that overturned the monarchy, and instituted a consular or republican form of government in its stead. There were five Brutuses. B. v., known to history as Junius Brutus, killed Julius Caesar because it was thought J. C. wanted to be king, and the Romans hated the very name of king; indeed they had sworn that there should never be another. After the death of Caesar, of course, every schoolboy knows that there came into power the long line of emperors. Thus these two Brutuses were the cause of two of the greatest events in European history—change of form of the Roman government from royalty to republican, and from republican to imperial. But no Brutus "discovered Britain" or named it.

But,—what's the use? When a man sets himself up as an authority and a teacher, he should be sure that he states facts. He should know his lesson. He should "be sure he is right and then go ahead," as the immortal Davy Crockett said. Dr. Bell should go back to his Virgil, Ovid and Livy. He should review Keightley, Gayley, Bullfinch, Tooke and Anthon, to say nothing of Strabo, Niebuhr, Cramer, et al. But,—what's the use?

## Figures and Tables

**Figure f1:**